# Modelling *Toxoplasma gondii* infection in human cerebral organoids

**DOI:** 10.1080/22221751.2020.1812435

**Published:** 2020-09-06

**Authors:** Hyang-Hee Seo, Hyo-Won Han, Sang-Eun Lee, Sung-Hee Hong, Shin-Hyeong Cho, Sang Cheol Kim, Soo Kyung Koo, Jung-Hyun Kim

**Affiliations:** a Division of Intractable Diseases, Center for Biomedical Sciences, Korea National Institute of Health, Korea Centers for Disease Control and Prevention, Cheongju, Republic of Korea; b National Stem Cell Bank of Korea, Korea Institute of Health, Cheongju, Republic of Korea; c Division of Vectors and Parasitic Diseases, Korea Centers for Disease Control and Prevention, Cheongju, Republic of Korea; d Division of Bio-Medical Informatics, Center for Genome Science, Korea National Institute of Health, Korea Centers for Disease Control and Prevention, Cheongju, Republic of Korea

**Keywords:** Cerebral organoid, pluripotent stem cells, Toxoplasma gondii, toxoplasmosis, disease modelling

## Abstract

Pluripotent stem cell-derived cerebral organoids have the potential to recapitulate the pathophysiology of *in vivo* human brain tissue, constituting a valuable resource for modelling brain disorders, including infectious diseases. *Toxoplasma gondii*, an intracellular protozoan parasite, infects most warm-blooded animals, including humans, causing toxoplasmosis. In immunodeficient patients and pregnant women, infection often results in severe central nervous system disease and fetal miscarriage. However, understanding the molecular pathophysiology of the disease has been challenging due to limited *in vitro* model systems. Here, we developed a new *in vitro* model system of *T. gondii* infection using human brain organoids. We observed that tachyzoites can infect human cerebral organoids and are transformed to bradyzoites and replicate in parasitophorous vacuoles to form cysts, indicating that the *T. gondii* asexual life cycle is efficiently simulated in the brain organoids. Transcriptomic analysis of *T. gondii*-infected organoids revealed the activation of the type I interferon immune response against infection. In addition, in brain organoids, *T. gondii* exhibited a changed transcriptome related to protozoan invasion and replication. This study shows cerebral organoids as physiologically relevant *in vitro* model systems useful for advancing the understanding of *T. gondii* infections and host interactions.

## Introduction

Toxoplasmosis is a parasitic disease caused by *Toxoplasma gondii*. It is a widespread disease in humans, and it has been estimated that approximately 30–50% of the world’s population is infected by this parasite [1]. The Felidae family is the only definitive host of *T. gondii*. These parasites undergo sexual development and reproduction in infected felines, eventually producing cysts containing formed zygotes [2].

Once *T. gondii* infects warm-blooded hosts, such as humans, it enters circulating cells, such as macrophages [3,4] and dendritic cells [4], enabling the parasites to cross the blood–brain barrier (BBB) within a few hours [5]. Subsequently, *T. gondii* preferentially forms bradyzoites, a chronic semi-dormant form of tachyzoites, and tissue cysts (containing bradyzoites) mainly in the brain [6,7]. Inside the cysts, several hundred bradyzoites remain hidden and protected from the host immune system. Thus, they can persist indefinitely in the host [8] and become the main sources of parasite transmission and pathogenesis, reactivating toxoplasmosis when the bradyzoites revert to tachyzoites [9,10]. In immunocompetent persons, toxoplasmosis is an asymptomatic infection. However, when immunodeficient patients, such as patients with AIDS or pregnant women, are infected, *T. gondii* can cause intracerebral mass lesions, retinochoroiditis, pneumonitis, and, occasionally, even death [11–13]. Congenital infection of *T. gondii* results in developmental defects in many organs. Brain tissue is one of the major organs found to have abnormalities because of multiplying parasites. Parasitic infection induces necrotic foci and profound inflammation, which may block the aqueduct of Sylvius and cause hydrocephalus of the lateral ventricles. These foci also calcify. The classic features related to brain developmental defects are mental retardation, seizures, microcephalus, hydrocephalus, cerebral calcifications, and abnormal cerebrospinal fluid [11,14].

More recently, many reports have suggested that there are unrecognized consequences in humans infected with *T. gondii*, *s*uch as impulsive and aberrant neurocognitive behaviour and psychiatric illnesses, including schizophrenia [15,16]. Although several drugs, including azithromycin, clarithromycin, spiramycin, atovaquone, dapsone and cotrimoxazole, have been used for the treatment of toxoplasmosis, but their effect in eliminating tissue cysts and bradyzoites has been unsatisfactory [17]. Further, there is no approved vaccine against toxoplasmosis [18,19].

The main challenge in the research of *T. gondii*-host interactions and drug development is the lack of an optimal *in vitro* model that could efficiently recapitulate *T. gondii* infection in humans. Several studies have used 2D cultures of microglia, astrocytes, and neuron cell lines as *T. gondii in vitro* model systems and demonstrated that these brain cell lines were susceptible to infection [20]. However, in most cases, the *T. gondii* infection was sustained for a short time and showed incomplete expansion [21]. In addition, the monolayer environmental condition is considerably different than that of a physiological 3D system, which may alter infectious processes, such as invasion, replication, and egression. A recent study showed that Vero cells in a 3D culture system showed a more consistent shape and proliferation pattern of *T. gondii* than was exhibited by a 2D culture system [21]. However, a 3D culture system based on a single cell type cannot fully recapitulate host–*T. gondii* interactions or lead to the determination of specific cell type-dependent host–parasite interactions [21].

Organoids have recently emerged as experimental tools for disease modelling, drug screening, regenerative medicine, and host–microbe interactions [22]. An organoid is a self-organizing stem cell-derived 3D structure consisting of organ-specific cells [23]. In contrast to monolayer culture systems, organoids resemble original tissues in terms of structural features, functionality, and genetic signatures [24]. By exploiting these similarities, we can perform research on diseases in organs and tissues that are not readily accessible because of their anatomic locations, such as the brain.

In the present study, we infected cerebral organoids with *T. gondii* tachyzoites and demonstrated that tachyzoites differentiated into bradyzoites, which replicated in parasitophorous vacuoles to form tissue cysts within the organoids. Our study sought to determine whether cerebral organoids infected with *T. gondii* recapitulate the human brain and can be used as model systems to understand *T. gondii*–host interactions.

## Materials and methods

### Cerebral organoid generation and culture

To generate cerebral organoids, we slightly modified the methods previously described by Lancaster *et al.* [25,26]. The H9 (WA09) human embryonic stem cell line, obtained from WiCell (Madison, WI, USA), was grown in mTeSR1 (Stem Cell Technologies, Vancouver, Canada) on a Matrigel-precoated plate (Corning, NY, USA), and the medium was changed every day. Colonies were detached from the Matrigel-coated plate using ReLeSR (Stem Cell Technologies), and 9000 cells were transferred to a round-bottom 96-well plate filled with AggreWell medium and 10% Clone R (Stem Cell Technologies). After 2 d, the medium was replaced with fresh AggreWell without Clone R, and the cells were incubated for an additional 3 d. On day 6, the medium was replaced with neural induction medium (NIM) containing DMEM/F12, N2 supplement (Thermo Fisher Scientific, Waltham, MA, USA), minimum essential medium-nonessential amino acids (MEM-NEAAs), GlutaMAX (Thermo Fisher Scientific) and 1 μg/ml heparin (Sigma, MO, USA), and the cells incubated for 5 d. On day 6, after the NIM medium was changed, embryoid bodies (EBs) were transferred by pipette onto Matrigel (Corning) droplets that were 3 mm in diameter. These droplets were incubated for 1 h at 37°C, transferred to 24-well plates and maintained in cerebral organoid differentiation medium (CORD) containing DMEM/F12, Neurobasal (Gibco), N2 supplement, NEAA (Gibco), an insulin solution (Sigma), the penicillin streptomycin (Sigma), GlutaMAX, β-mercaptoethanol (Gibco) and B-27 (without vitamin A) (Thermo Fisher Scientific) for 3 d. After 3 d, the medium was replaced with cerebral organoid differentiation medium containing vitamin A (CORDA), DMEM/F12, Neurobasal, N2 supplement, NEAA, an insulin solution, GlutaMAX, β-mercaptoethanol, and B-27 (with vitamin A) (Gibco) and maintained on a spinning bioreactor (N-BIOTEK). The medium was replaced every 3 d.

### Toxoplasma gondii culture

Tachyzoites of the *T. gondii* PTG-GFP 5 S65 T, haplogroup 2, ME49 strain (50941, ATCC, Manassas, VA, USA) and those of the *T. gondii* RH-GFP strain were maintained *in vitro* using Vero cells (CCL-81, ATCC) cultured in DMEM (Gibco) supplemented with 10% FBS (Gibco) at 37°C in a humidified incubator with an atmosphere of 5% CO_2_. *T. gondii* were allowed to replicate for 3 d in Vero cells homogenized with 1 mL of Dulbecco's phosphate-buffered saline (Gibco/Life Technologies, Grand Island, NY, USA) using a 23-gauge needle. The tachyzoites of the *T. gondii* RH strain that expresses transgenic green fluorescent protein (RH-GFP) were provided by Dr. Yoshifumi Nishikawa (Obihiro University of Agriculture and Veterinary Medicine, Japan).

### Organoid infection and live imaging

Initially, the medium in which the cerebral organoids was maintained was removed, and pre-chilled Cell Recovery solution (Corning) was added. Using a wide-bore tip, the solution was pipetted up and down, and the Matrigel was carefully removed to avoid damaging the organoids. Then, the cell culture plates were incubated at 4°C for 20 min. When the organoids were free from the Matrigel, they were washed three times with PBS and then fresh media were added. Next, 1 × 10^3^ ME49 tachyzoites and RH tachyzoites were incubated with separate cerebral organoids, each in a well with 200 μl of culture medium, in a 96-well plate for 4 h at 37°C in a humidified incubator with an atmosphere of 5% CO_2_. After 4 h, the cerebral organoids were washed three times with PBS and fresh medium was added.

For live-cell imaging, GFP-tagged *T. gondii*-infected organoids were transferred to a 35-mm dish, and fresh medium was added. The organoids were maintained at 37°C in an automated microscope chamber with a humidified atmosphere of 5% CO_2_ (BioTek Lionheart FX, BioTek Instruments, Winooski, VT, USA). Time-lapse images were acquired every 30 min for a total of 50 h. In total, nine positions were imaged, and a z-stacking process was performed.

### RNA isolation and RNA-seq analysis

RNA was extracted using the RNeasy Plus mini kit (Qiagen, Hilden, Germany) according to the manufacturer’s protocol. The library was prepared using the TruSeq Stranded mRNA LT Sample prep kit (Illumina, San Diego, CA, USA) and run on a HiSeq 4000 sequencer (Illumina) with a 101-bp paired-end read length. Raw data were processed using HISAT2 [27] to align genome reads with reference genomes (hg19 for humans and GCF_000006565.2_TGA4 for the ME49 strain of *T. gondii*).

### Histology and immunofluorescence staining

Cerebral organoids were fixed overnight in 4% paraformaldehyde at 4°C. The following day, the Matrigel was removed, and the organoids were incubated in fresh 4% paraformaldehyde for 1 h at 4°C. Subsequently, the organoids were allowed to sink in a 30% sucrose solution at 4°C and then were embedded in optimal cutting temperature (OCT) compound (Scigen, Gardena, CA, USA) and incubated at −80°C for 1 d. Thereafter, the organoids were cryosectioned into 20-μm-thick sections, permeabilized in 0.3% Triton X-100, and blocked with a 3% bovine serum albumin solution for 1 h. The sections were then incubated overnight with primary antibodies at 4°C. The following antibodies were used in this study: anti-SOX2 (AB5603, Sigma), anti-PAX6 (MA1-109, Invitrogen, Carlsbad, CA, USA), anti-SATB2 (ab34735, Abcam), anti-O4 (MAB1326, R&D system, MN, USA), anti-O1 (MAB1327, R&D system), anti-GFAP (ab7260, Abcam) anti-DAPI (D1306, Invitrogen), and anti-TUJ1 (801202, BioLegend, San Diego, CA, USA). The following secondary antibodies were purchased from Invitrogen: Alexa Fluor 594 (A21207 and A21203) and Alexa Fluor 488 (A21206 and 21202). Immunofluorescence was detected by confocal microscopy (LSM800; Carl Zeiss, Oberkochen, Germany), and analysis was performed using ZEN 2 blue edition software (Carl Zeiss).

### Ingenuity pathway analysis (IPA)

To predict the possible diseases and disorders in the ME49- and RH-infected cerebral organoids, differentially expressed genes were evaluated by ingenuity pathway analysis (IPA) (Ingenuity Systems, Redwood City, CA, USA). A *p*-value of less than 0.05 was considered significant.

### Enzyme-linked immunosorbent assay (ELISA) for T. gondii

Serum obtained from C57BL/6 mice infected with *T. gondii* tachyzoites was assayed by enzyme-linked immunosorbent assay (ELISA) using antibodies against the *T. gondii* P30 protein with a Multispecies ID Screen® Toxoplasmosis Indirect kit (IDVET, Montpellier, France) following the manufacturer’s instructions. The serum was diluted 1:9 with dilution buffer and incubated for 1 h at room temperature. The plates were rinsed three times with washing buffer, after which horseradish peroxidase (HRP)-conjugated goat anti-mouse IgG antibody (Sigma, St. Louis, MO, USA) was added, and then, the plates were incubated for 1 h at room temperature. After the final washing, substrate solution was added to each well and incubated for 15 min at room temperature in the dark. The reaction was stopped using 0.5 M H_2_SO_4_ solution, and the absorbance was read at 450 nm using an ELISA reader. Mean OD values > 0.5 were defined as positive [28].

### Transmission electron microscopy

Organoids were primarily fixed overnight with Karnovsky’s fixative (2% glutaraldehyde and 2% paraformaldehyde in 0.5 M cacodylate buffer, Electron Microscopy Sciences, Hatfield, PA, USA) at 4°C and post fixed with 1 M osmium tetroxide (diluted in 0.1 M sodium cacodylate buffer). After dehydration in a series of gradient ethanol solutions, the samples were subjected to propylene oxide and then polymerized using Spurr’s resin (Electron Microscopy Sciences, Hatfield, PA, USA). The samples were subsequently cut into 70-nm-thick slices and loaded onto a 2 × 1 mm CU grid coated with Formvar/carbon film (Electron Microscopy Sciences, Hatfield, PA, USA). Transmission electron microscopy (TEM) images were obtained using a TEM microscope (80 kV) (JEOL, TYO, JAPAN).

### Statistical analysis

The quantitative data are presented as the means ± S.E.M. based on at least 3 independent experiments. For statistical analysis, Student’s t-test was used to compare two experimental groups, and for comparisons of more than 3 groups, one-way ANOVA with Bonferroni correction was performed with GraphPad Prism 5.0 (GraphPad Software, Inc., San Diego, CA). A *p*-value of less than 0.05 was considered statistically signiﬁcant.

## Results

### *In vitro* generation of cerebral organoids from human embryonic stem cells

**Figure 1.**
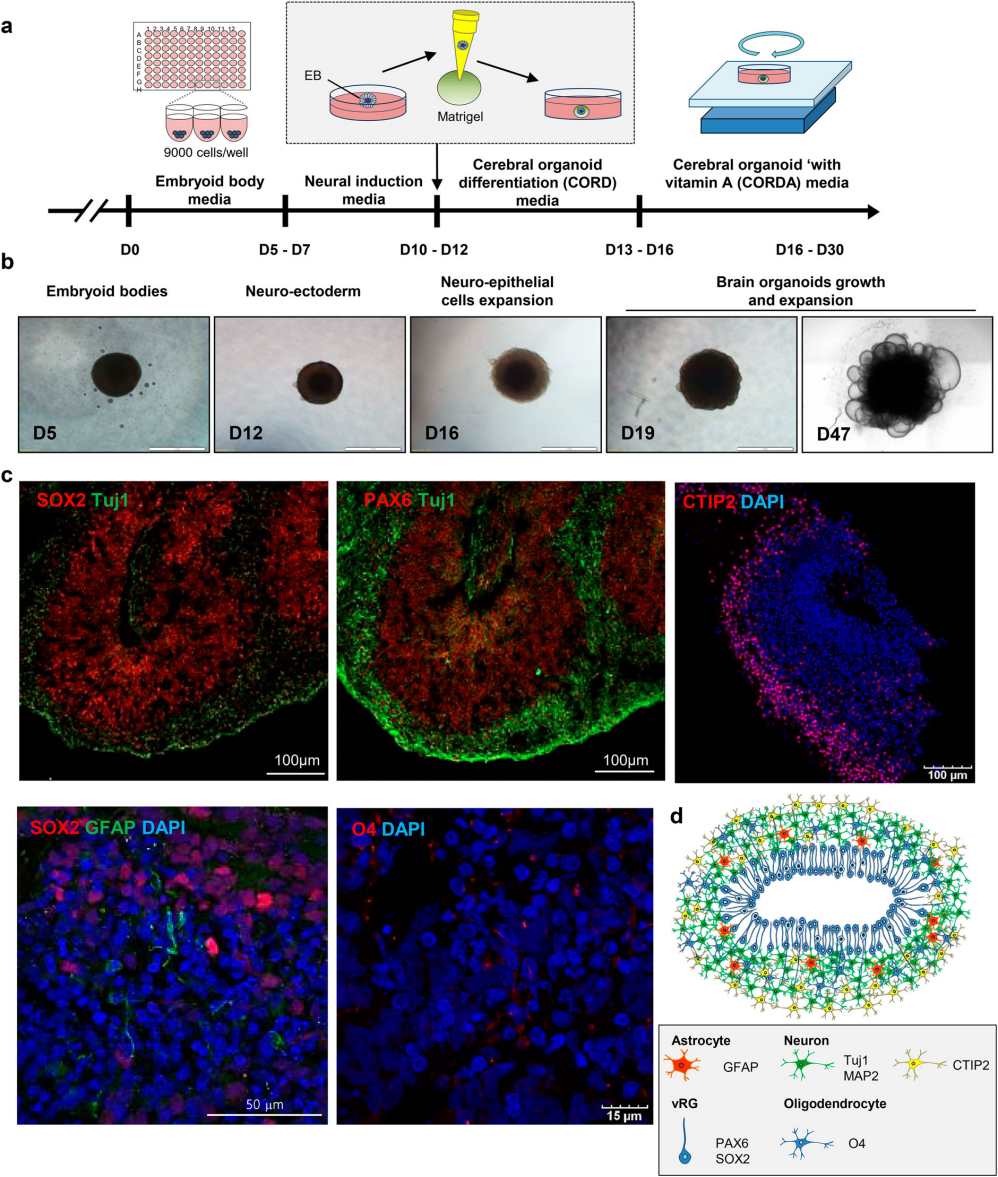
Generation of cerebral brain organoids. (A) Schematic showing the method for generating hESC-derived brain organoids. (B) Representative images of a developing cerebral organoid at specific time points. (C) Immunohistochemistry of markers for the detection of neurons (SOX2 and TUJ1), radial glial cells (PAX6), astrocytes (GFAP) and oligodendrocytes (O1 and O4). Scale bars, as indicated. (D) Schematic representation of cerebral organoids generated in this study.

To generate cerebral organoids, we modified the previously reported methods described by Lancaster *et al.* [25,26]. Briefly, we seeded 9000 cells in each round-bottom well of a 96-well plate to generate uniformly sized embryoid bodies (EBs). After 5–7 d, we confirmed that the EBs were of similar sizes (Fig. S1A and S1B). Next, the EBs were maintained in neural induction medium for 5 d to generate neuroectodermal tissue, and a Matrigel droplet was embedded into each well to provide a scaffold. Plate cultures containing these Matrigel droplets were then placed on an orbital shaker to increase oxygen-nutrient absorption (Figure 1A, B and S2). To test whether cerebral organoids mimic key structural features of the human brain, histological analysis was performed. On day 50 of differentiation, cerebral organoids were stained for specific markers of a diverse set of brain cell types. We observed bipolar-shaped SRY-Box 2 (SOX2)^+^/paired box 6 (PAX6)^+^/progenitor cell populations in the ventricular zone. In addition, a large number of cells expressing the lower-layer cortical neuronal marker COUP TF1-interacting protein 2 (CTIP2) were detected as cortical plate (CP)-like structures. Neuron-specific class III beta-tubulin (TUJ1)+ neuronal layer GFP-positive cells were also found in the highly proliferating neuronal cell layer (Figure 1C). Furthermore, on day 55, glial fibrillary acidic protein (GFAP)^+^ astrocytes and O4^+^ oligodendrocytes started to appear, and by day 70, a large number of GFAP+ and O4+ cells were observed in the cerebral organoids (Figures 1C, S3 and S4).

### Infection of human cerebral organoids with *T. gondii*

**Figure 2.**
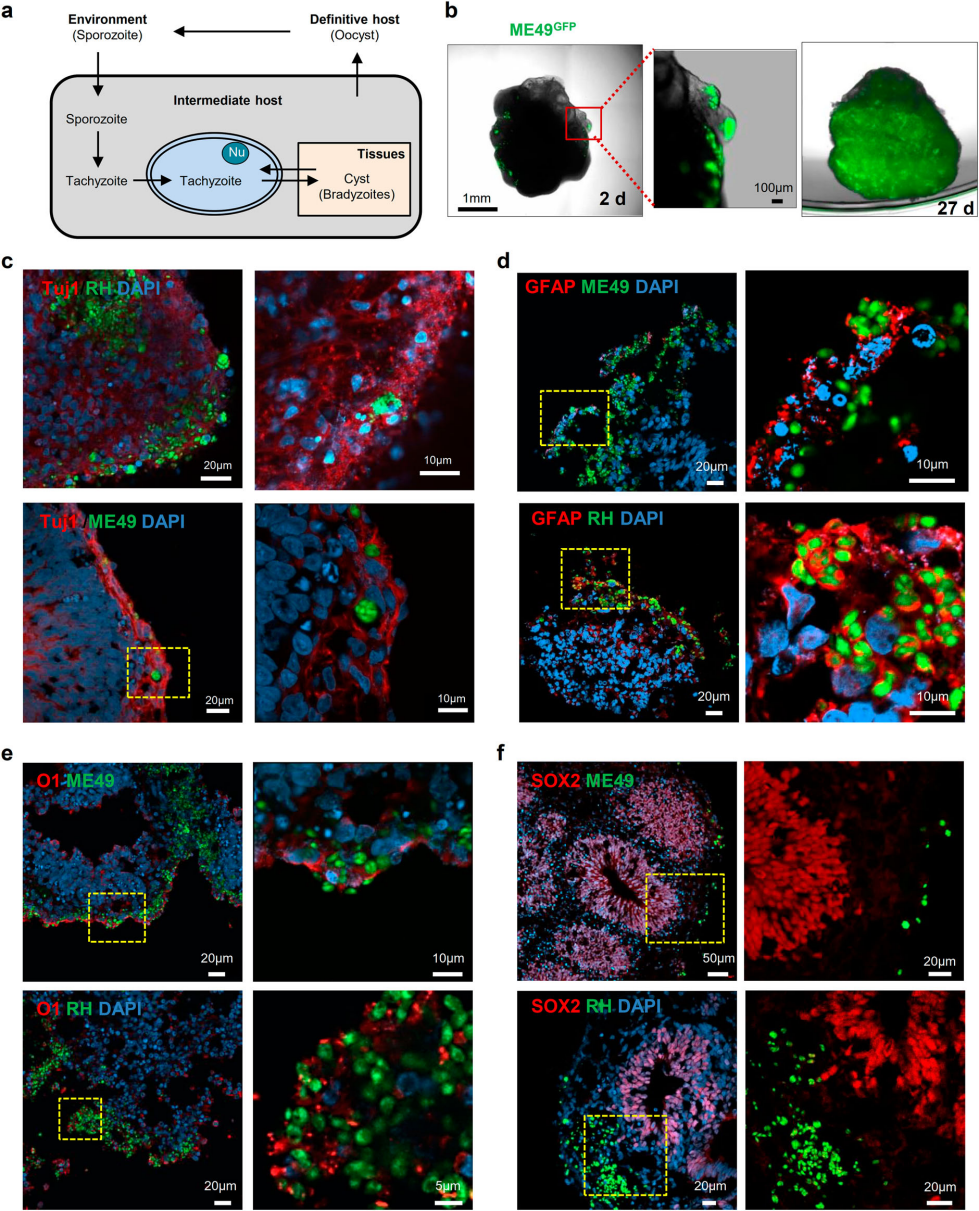
Distribution of *Toxoplasma gondii* in the human cerebral organoids. (A) Schematic representation of the life cycle of *T. gondii*. (B) 3D images of a cerebral organoid infected with *T. gondii* (green). (C – F) Representative fluorescence images of cerebral organoids infected with 2 strains of *T. gondii*: ME49 (top) and RH (bottom) infected cerebral organoids are shown stained for (C) TUJ1, a neuronal marker; (D) GFAP, an astrocyte marker; (E) O1, an oligodendrocyte marker; and (F) SOX2, a radial glial cell marker. Scale bars, as indicated.

Although *T. gondii* infection has been reported in 2D cell cultures, the transformation of *T. gondii* tachyzoites to bradyzoites, which is a central event of chronic toxoplasmosis infection (Figure 2A) and constitutes a life-long risk for recurring infection [11], has not been studied *in vitro*. Thus, we sought to determine whether cerebral organoids can be used as an *in vitro* models for *T. gondii* infection. To investigate whether parasites entered and were sustained in the human cerebral organoids, tachyzoites from two *T. gondii* strains, the type I (RH) strain expressing green fluorescent protein (RH-GFP) and the type II strain (ME49) also expressing green fluorescent protein (ME49-GFP), were incubated with cerebral organoids for 4 h. As shown in Figure 2B, GFP signals were detected within the organoids 2 days post-infection in the ME49-infected cerebral organoids. To study this signalling in more detail, we set up a live-imaging experiment using fluorescent microscopy of cerebral organoids 1 d after ME49 infection (Movie S1). We initially observed that the *T. gondii* cells invaded the surface of the organoid. However, by day 27, the parasites had propagated within the cerebral organoid, as shown by the GFP-positive signals throughout the cerebral organoid (Figure 2B).

Next, we examined whether *T. gondii* preferentially infects specific brain cells, such as neuronal cells, neuronal progenitor cells, astrocytes, oligodendrocytes. ME49-GFP infected (CO^ME49^) and RH-GFP infected (CO^RH^) cerebral organoids were fixed at 3 days post-infection and stained for cell-type specific markers. We found that *T. gondii* (green) was co-detected with TUJ1, a neuronal marker; GFAP, a marker of astrocytes; and O1, a marker of oligodendrocytes. These findings indicated that the ME49 and RH strains preferentially infected these cell types in the cerebral organoids (Figure 2C, D and E). In contrast, none of the *T. gondii* strains colocalized with SOX2, a marker of radial glial cells (Figure 2F).

### *T. gondii* form cysts in cerebral organoids

**Figure 3:**
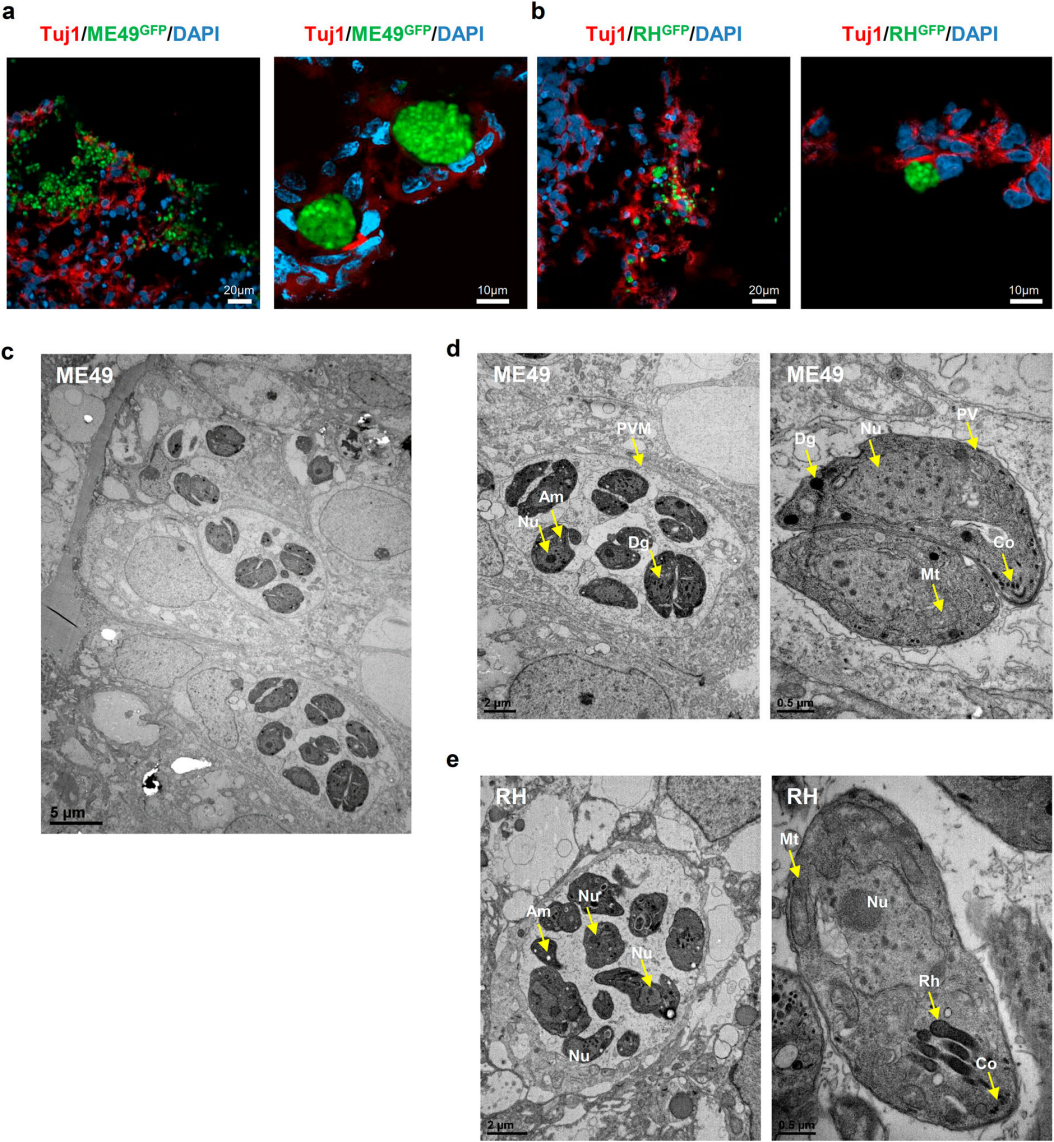
. *T. gondii* cyst formation in human cerebral organoids. Representative fluorescence image of cyst-like structures in an organoid infected with (A) ME49 and (B) RH. Images of transmission electron microscopy of (C–D) ME49- and (E) RH-infected cerebral organoids. Scale as indicated with the bar. PVM, parasitophorous vacuole membrane; Nu, nucleus; Rh, rhoptry; Co, conoid; Mt, mitochondrion; Dg, dense granule; and Am, amylopectin.

In intermediate hosts, *T. gondii* can form tissue cysts (bradyzoites), which persist throughout the life of the host [11]. In addition, bradyzoites in tissue cysts can dedifferentiate into tachyzoites, representing the replicative stage (Figure 2A). To address whether *T. gondii* can replicate within the organoids, we first examined the cyst-like formations of densely packed *T. gondii* in the cerebral organoids. We observed that by 5 days post-infection, both *T. gondii* strains had formed tissue cyst-like structures within the cerebral organoids enveloped by TUJ1-positive cells (Figure 3A and B). To ascertain the developmental stages of these *T. gondii* in the organoids, we performed TEM imaging and observed a diverse number of bradyzoites, which were characterized by the presence of amylopectin granules (Am) located inside the parasitophorous vacuole membranes (PVMs). This observation indicates that *T. gondii* shows interconversion between the rapidly growing tachyzoite and latent encysted bradyzoite stages, recapitulating the complex life cycle stages of the parasite within the organoids (Figure 3C, D, and E).

### *T. gondii* exhibit virulence in cerebral organoids

**Figure 4.**
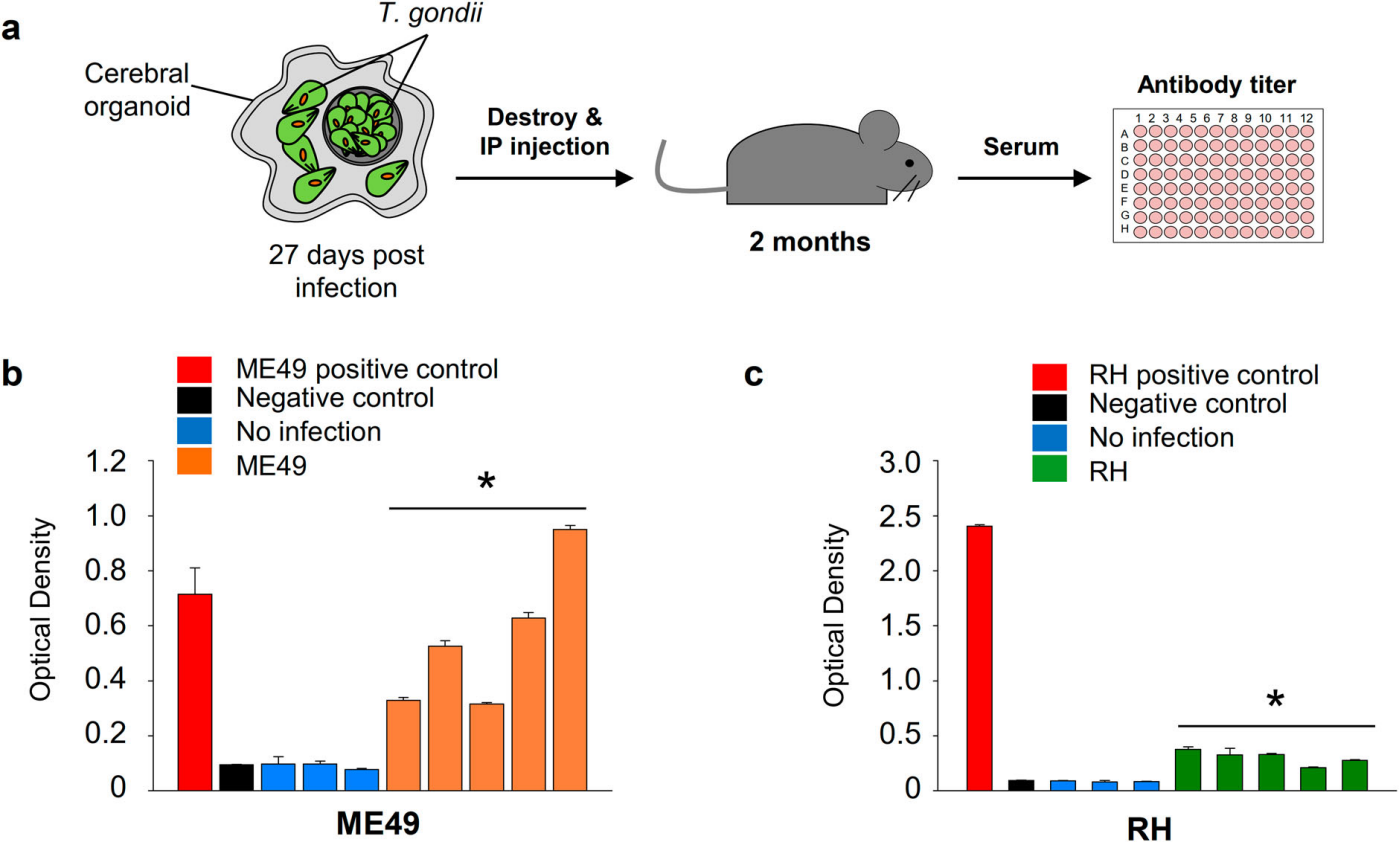
Virulence of *T. gondii* in the infected cerebral organoids**.** (A) Schematic representation of the experimental design: *T. gondii* isolated from infected cerebral organoids was injected into mice. The levels of *T. gondii* P30 protein were measured by ELISA (*n* = 5, biologically independent mice) 2 months postinfection. (B) ME49 and (C) RH antibody titre presented as the optical density of ELISA . *p < 0.05. Quantitative data are expressed as the mean ± S.E.M. of at least 3 independent experiments.

We evaluated whether *T. gondii* parasites in infected organoids remained virulent. To examine *T. gondii* virulence in the organoids, we mechanically dissociated infected organoids and introduced them into mice through intraperitoneal injection. After two months, mouse serum was collected, and antibodies against the *T. gondii* P30 protein were measured (Figure 4A). We observed a 3.4- to 10.4-fold (*p*-value < 0.05) increase in the level of *T. gondii* P30 protein in the CO^ME49^-injected mice and a 2.4- to 4.4-fold (*p*-value < 0.05) increase in the CO^RH^-injected mice, compared to the noninfected mice (Figure 4B and C).

### Transcriptomic analyses of *T. gondii* and infected organoids

**Figure 5.**
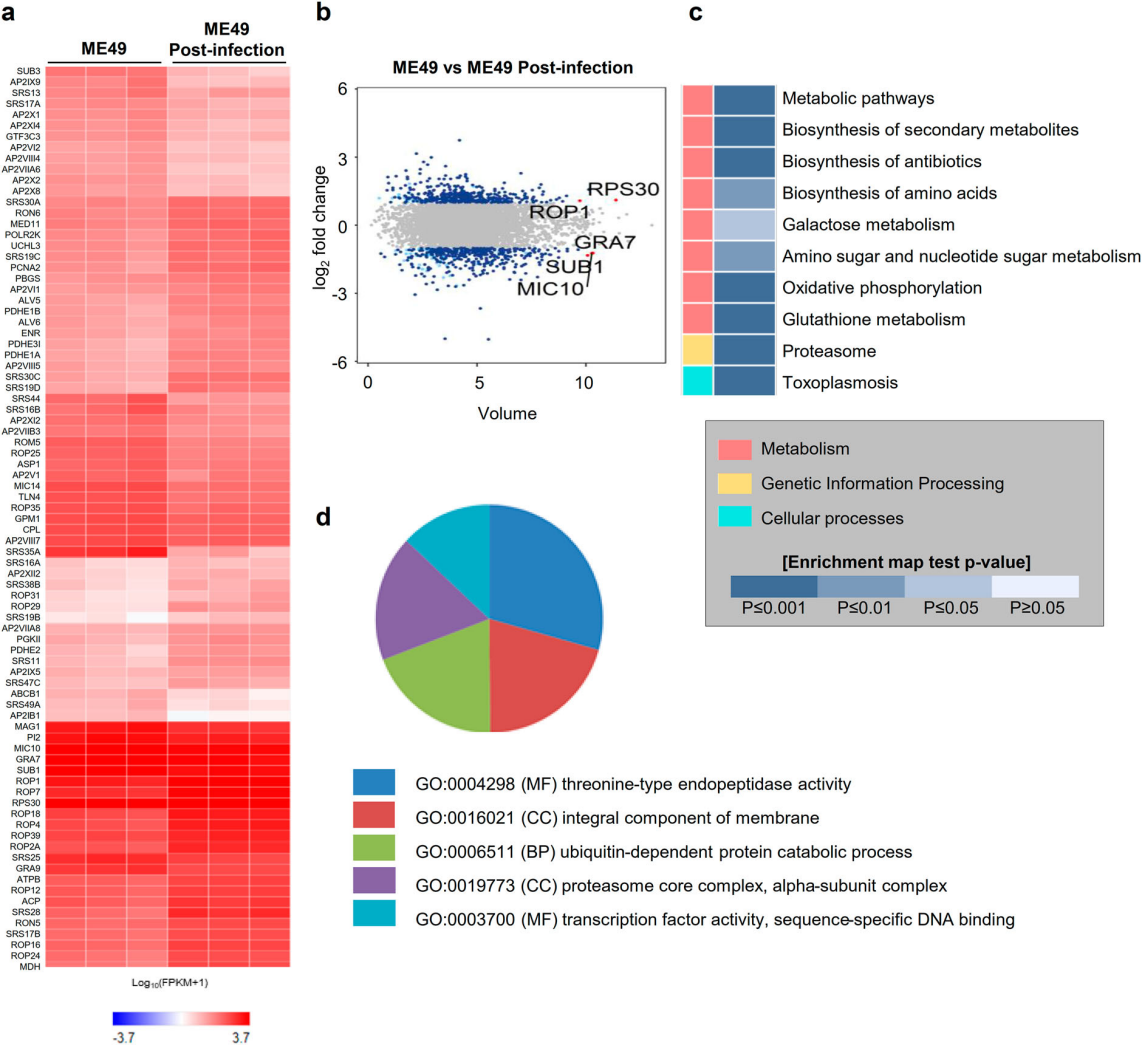
Transcriptome analysis of ME49 post-infection. (A) Differentially expressed genes of ME49 post-infection. Noninfectious ME49 was used as a control (|fc| ≥ 2, raw *p*-value < 0.05). A heat map was generated using Cluster grammer (http://amp.pharm.mssm.edu/clustergrammer/). (B) Volume plot showing differential expression of ME49 genes between 0 and 72 h post-infection (|fc| ≥ 2, raw *p*-value < 0.05). The top 5 gene names are indicated. (C) KEGG pathway analysis was performed using the differentially expressed genes (DEGs) of ME49 (|fc| ≥ 2, raw *p*-value < 0.05). (D) Gene Ontology (GO) annotation of *T. gondii* postinfection of cerebral organoids; *p*-value < 0.01. MF, molecular function; BP, biological process; and CC, cellular component.

Next, we performed a transcriptome-sequencing analysis of the ME49 strain of *T. gondii* 0 and 72 h post-infection. Hierarchical clustering of the differentially expressed genes (DEGs) (|fc| ≥ 2, raw *p*-value < 0.05) showed that three independent ME49 genes were clustered separately at 0 and 72 h post-infection (Fig. S5). We identified 786 genes, of which 402 genes were upregulated and 384 genes were downregulated, 72 h post-infection. (Figure 5A and B, Fig. S5, Table S1; |fc| ≥ 2, raw *p*-value < 0.05). We found that ribosomal proteins, such as ribosomal protein S20 (RPS20), S14 (RPS14), L15 (RPL15), and S14 precursors, which are generally expressed in bradyzoites [29], were highly expressed in the ME49 strain 72 h post-infection, indicating bradyzoite formation in the brain organoids. Moreover, rhoptry organelle proteins (ROPs) were elevated in the post-infected ME49 strain, indicating the potential ME49 invasion and establishment of the replicative niche in the parasitophorous vacuoles in the brain organoid cells [30,31]. Subsequently, Kyoto Encyclopedia of Genes and Genomes (KEGG) pathway and Gene Ontology (GO) analyses were conducted on the DEGs from 72 h post-infected ME49 strain. Toxoplasmosis was predicted by the KEGG analysis, suggesting that transcriptomic changes to the ME49 strain in the infected brain organoids recapitulated toxoplasmosis (Figure 5C). The GO analysis predicted that both threonine-type endopeptidase activity-related processes and proteasome core complexes, which are involved in host cell invasion of apicomplexan parasites, particularly *T. gondii* [32], would be increased in the ME49 strain in the infected brain organoids compared to their predicted occurrence in tachyzoites (Figure 5D).

**Figure 6:**
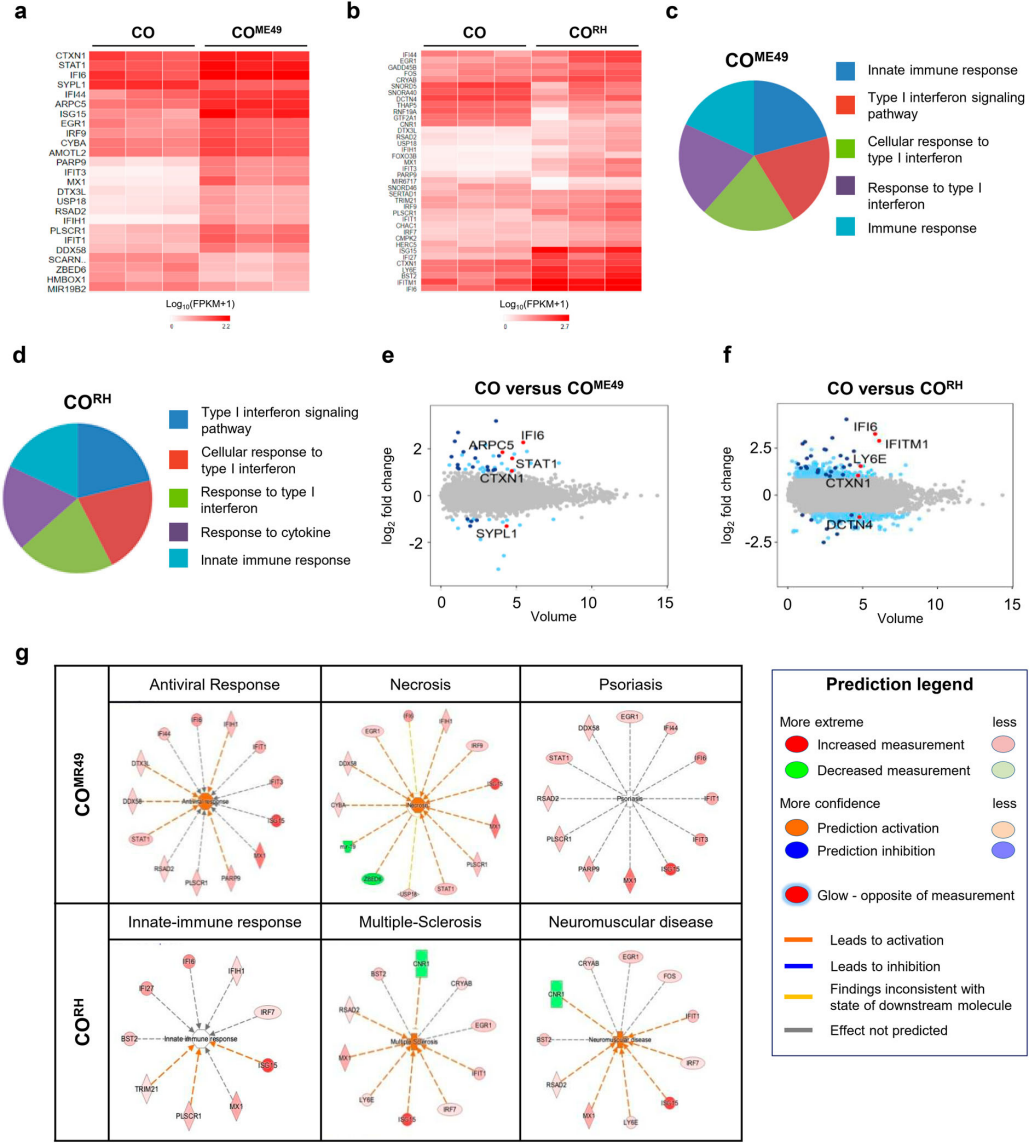
. Transcriptome analysis of *T. gondii*-infected cerebral organoids. Heat map showing differentially expressed genes (DEGs) of (A) ME49- and (B) RH-infected cerebral organoids 72 h post-infection (|fc| ≥ 2, raw *p*-value < 0.05). Noninfected cerebral organoids were used as controls (*n* = 3, biologically independent samples). A heat map was generated using Cluster grammer (http://amp.pharm.mssm.edu/clustergrammer/). Gene Ontology analysis of DEGs in the (C) ME49- and (D) RH-infected organoids. Top five Gene Ontology terms (*p*-value < 0.005). Volume plot showing the top five genes (marked as red dots) in the (E) ME49- and (F) RH-infected organoids. Significantly upregulated and downregulated genes (|fc| ≥ 2, raw *p*-value < 0.05) are marked in blue. Noninfected organoids were used as controls. (G) Transcriptome data were analyzed using ingenuity pathway analysis (IPA) software (*p*-value < 0.05).

Next, we performed transcriptomic analysis of the cerebral organoids after 72 h of *T. gondii* infection. The heat map of the one-way hierarchical clustering using z-scores for normalized values (|fc| ≥ 2, raw *p*-value < 0.05) showed that all three independent samples of CO, CO^ME49^, and CO^RH^ (Fig. S6A and S6B) were clustered together. Our results revealed that 25 and 38 DEGs were identified in the CO^ME49^ and CO^RH^, respectively (Figure 6A and B). Type I IFN contributes to the modulation of chronic *T. gondii* persistence in the central nerve systems [33,34]. Our study supported the observation that the type I IFN signalling pathway was highly altered by *T. gondii* infection in both of the strains analyzed by GO (Figure 6C and D). We additionally identified the top five DEGs in both the CO^ME49^ (interferon alpha-inducible protein 6 (IFI6), signal transducer and activator of transcription 1 (STAT1), cortexin-1 (CTXN1), actin-related protein 2/3 complex subunit 5 (ARPC5), and synaptophysin-like 1 (SYPL1)) and CO^RH^ (IFI6, interferon-induced transmembrane protein 1 (IFITM1), lymphocyte antigen 6 family member E (LY6E), CTXN1, and dynactin subunit 4 (DCTN4)) models (Figure 6E and F; |fc| ≥ 2, raw *p*-value < 0.05) were related to type 1 IFN signalling. Next, to validate the activation of the type I IFN signalling pathway during *T. gondii* infection, we conducted qPCR analysis. The expression of the representative genes of the type I IFN signalling pathway (IL-10, TNF-α, IFN-α, and ISG15) was increased in the *T. gondii*-infected organoids (Fig. S7). Subsequently, we applied ingenuity pathway analysis (IPA) to predict the functions and disease outcomes in the cerebral organoids following *T. gondii* infection. The top three infection outcomes identified in the CO^ME49^ were antiviral response, necrosis, and psoriasis, whereas the top three outcomes in the CO^RH^ were innate-immune response, multiple-sclerosis, and neuromuscular disease (Fig. 6G).

## Discussion

In this study, we evaluated the possibility of using human embryonic stem cell-derived cerebral organoids as an *in vitro* model systems for studying *T. gondii* infections. Until now, the research on *T. gondii* infection has highly relied on 2D cell culture conditions or studies on animals, such as rodents. Although one study has been reported regarding the study of *T. gondii* infection in a 3D cell line culture system consisting of a collagen matrix, the model was unable to recapitulate the tissue complexity in terms of diverse cell types, conditions and interactions between the host and the parasites [21].

Here, we generated cerebral organoids and infected them with two GFP-tagged *T. gondii* strains (ME49^GFP^ and RH^GFP^) to assess whether our *T. gondii*-infected cerebral organoids could recapitulate the *in vivo* human brain system. Accordingly, we investigated the distribution of *T. gondii* within the organoids. The immunofluorescence data revealed that *T. gondii* showed mainly preferential targeting to neuronal cells, astrocytes, and oligodendrocytes but not to radial glial cells in the cerebral organoid culture system. *T. gondii* infects nucleated cells, such as neurons, astrocytes, and glial cells, in rodents [35,36]. Despite this recapitulation, the cell type sensitivity of the *T. gondii* infection has not been sufficiently characterized in toxoplasmosis patients or in 3D human cell culture systems. One recent study reported on the prevalence of *T. gondii* in a series of human autopsy samples, and the authors stated that *T. gondii* was detected in the neurons and astrocytes in the brains of these toxoplasmosis patients [37], findings consistent with our data. Regarding radial glial cells, one report demonstrated that *T. gondii* can infect rodent radial glial cells [38]; however, the considerable differences between humans and other species is notable. To date, it is not known whether human radial glial cells are susceptible to *T. gondii*. However, *T. gondii* infection models of brain organoids provided evidence suggesting that radial glial cells are not the main targeted cell type, with *T. gondii* displayed higher preferentiality infecting differentiated neuronal cells. Given the lack of data on human cells, we can only speculate that the observed lack of *T. gondii* infectivity in radial glial cells is due to a species-type-dependent effect. However, to verify the mechanism by which radial glial cells escape *T. gondii* infection in 3D culture systems and to quantify the selective tropism of *T. gondii* in cerebral organoids, further studies are required.

Next, we aimed to show the formation of tissue cyst-like structures in *T. gondii* in the cerebral organoids. Using immunofluorescence staining, we observed densely packed bradyzoites located in the neuronal cells of the cerebral organoids. Our TEM images of an organoid infected with tachyzoites also revealed the presence of parasitophorous vacuoles (PV) surrounding intracellular tachyzoites and cyst-like structures. Because tachyzoites can differentiate into bradyzoites in the host cell and form tissue cysts [37], we speculated that *T. gondii* efficiently simulated its asexual life cycle and converted between its tachyzoite and bradyzoite stages in the cerebral organoids. Moreover, the *T. gondii* strains isolated for the present study showed similar immunological responses in mice [39]. Of immunological interest, although the RH strain has high virulence, the antibodies in the CO^ME49^-injected mice were higher than they were in the CO^RH^-injected mice. However, the reason for the immunological difference was not found, and may be due to differences in the P30 protein. Further studies to determine immunological differences between the two strains should be conducted in a brain organoid 3D culture system.

RNA-sequencing data revealed the host–pathogen interaction responses of both *T. gondii* strains examined in infected human cerebral organoids. Despite the two different strains by which the cerebral organoids were infected, we observed that, in both cases, type I interferon (IFN-I) was an important cytokine in the immune response to the parasitic infection [40]. Interferon is an important cytokine in innate immune responses to parasitic infections. In *T. gondii*-infected mice, IFN-I was detected in the serum, spleen, and brain, and IFN-I expression gradually increased as the infection progressed [41–44]. One of the limitations of the cerebral organoids in this study was caused by the lack of innate immune cells, such as microglia and dendritic cells (DCs). In vivo studies have revealed that the proinflammatory cytokine IL-12 is stimulated by DCs and macrophages, thus inducing natural killer (NK) and NK T cells to release IFN gamma, resulting in the activation of antimicrobial machinery. In addition, DCs and macrophages present parasite antigens and costimulatory molecules that subsequently prime T cells [31]. According to the IPAs based on the DEGs and downstream effects in *T. gondii*-infected cerebral organoids, type I interferon, antiviral and innate immune responses were detected. However, IL-12-related immune responses were not detected in either the ME49- or RH-infected cerebral organoids. To improve the model system, the presence of major innate immune cells during organoid development is necessary.

Interestingly, cerebral organoids infected with the RH strain showed an increase in factors associated with multiple sclerosis (MS). It was reported that *T. gondii* and MS were negatively correlated [45]. However, *Yaman et al*. suggested that exposure to *T. gondii* may be one of the causes of MS development [46]. These contradicting reports indicate that the relationship between *T. gondii* and MS remains controversial. Our results implied that modelling *T. gondii* infection in cerebral organoids may allow for the investigation in-depth mechanisms of specific diseases related to *T. gondii* infection.

In summary, in this study, we evaluated the possibility of disease modelling for *T. gondii* infection using human cerebral organoids. We observed that tachyzoites were able to differentiate into bradyzoites and form cyst-like structures, indicating that the asexual life cycle and stage conversion of *T. gondii* were effectively simulated in our organoid culture system. We also revealed that a specific strain does not preferentially infect the brain. *T. gondii* transcriptomic analysis revealed gene expression changes upon organoid infection with *T. gondii*, which enabled the prediction of the biological process of *T. gondii* in the human brain organoids. In conclusion, we believe that the 3D cerebral organoids can be physiologically relevant model systems useful for understanding *T. gondii* infection.

## Supplementary Material

Supplemental Material

Figure_S7.tif

Figure_S6.tif

Figure_S5.tif

Figure_S4.tif

Figure_S3.tif

Figure_S2.tif

Figure_S1.tif

Movie_S1.mp4
