# Supplementary figures and images for: Modelling Toxoplasma gondii infection in human cerebral organoids
Source: Emerg Microbes Infect. 2020 Sep 6;9(1):1943–54. doi: 10.1080/22221751.2020.1812435 (PMC7534270; doi:10.1080/22221751.2020.1812435)

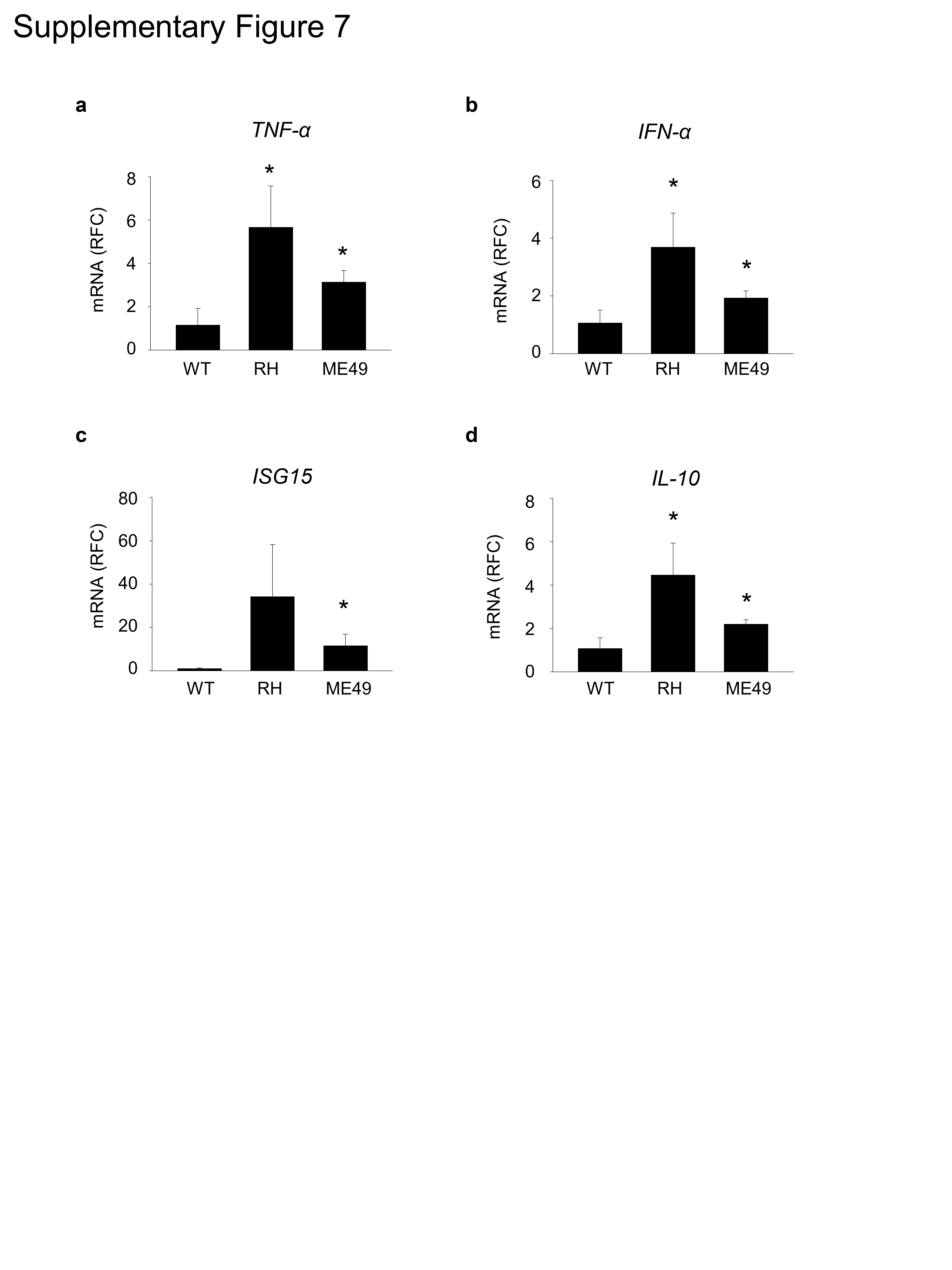

Supplement: Figure_S7.tif [file TEMI_A_1812435_SM2975.tif]

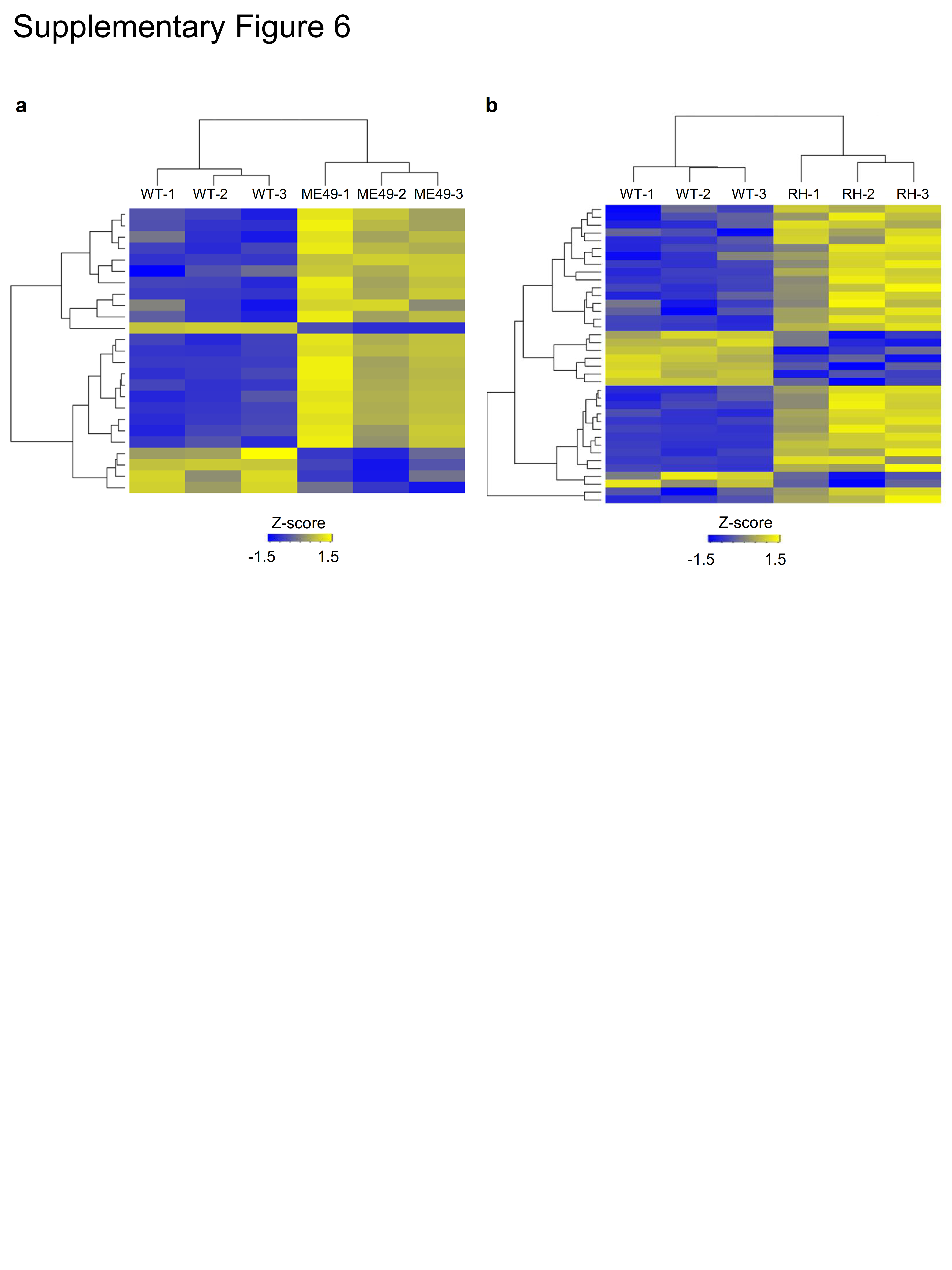

Supplement: Figure_S6.tif [file TEMI_A_1812435_SM2974.tif]

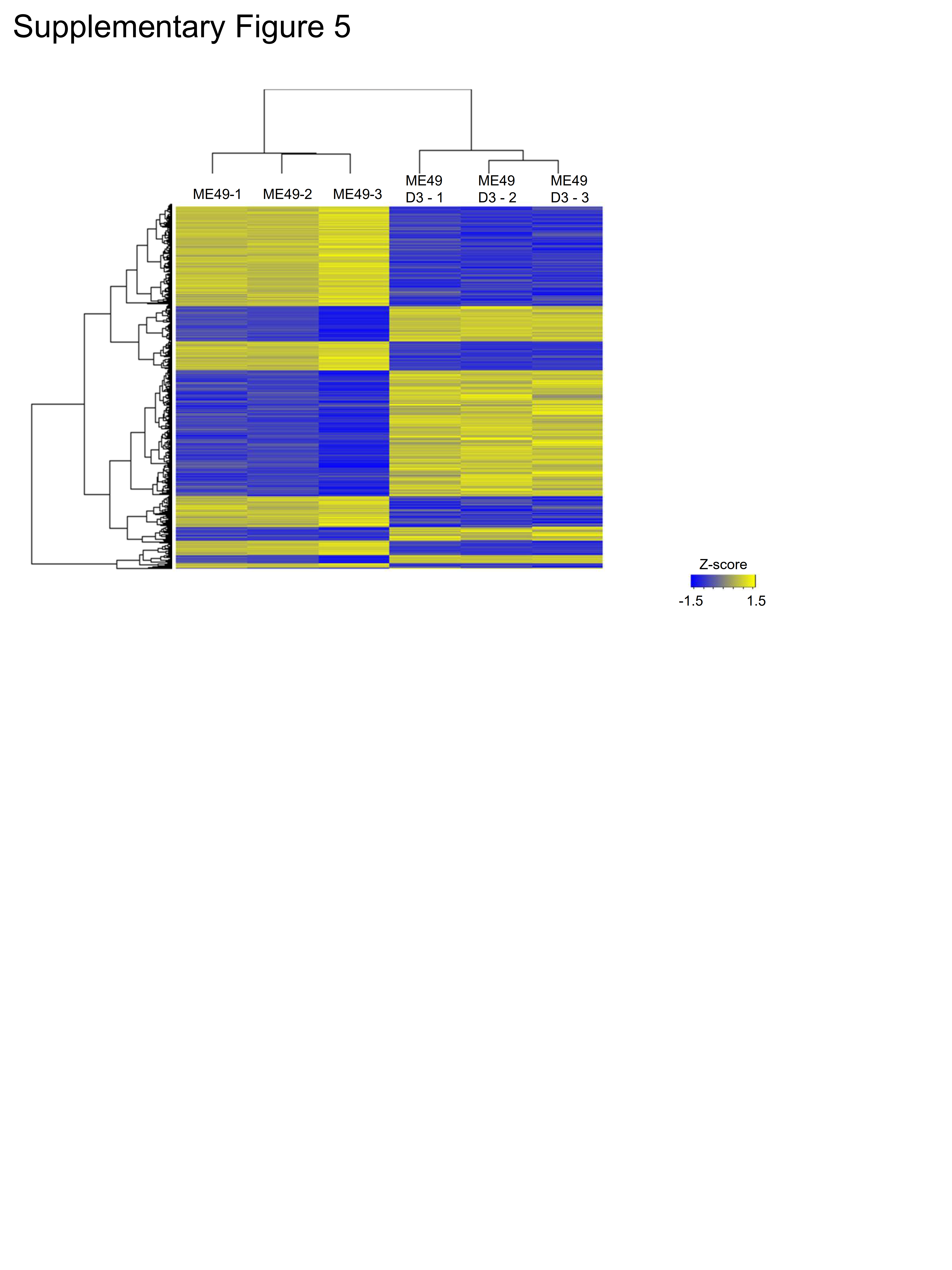

Supplement: Figure_S5.tif [file TEMI_A_1812435_SM2973.tif]

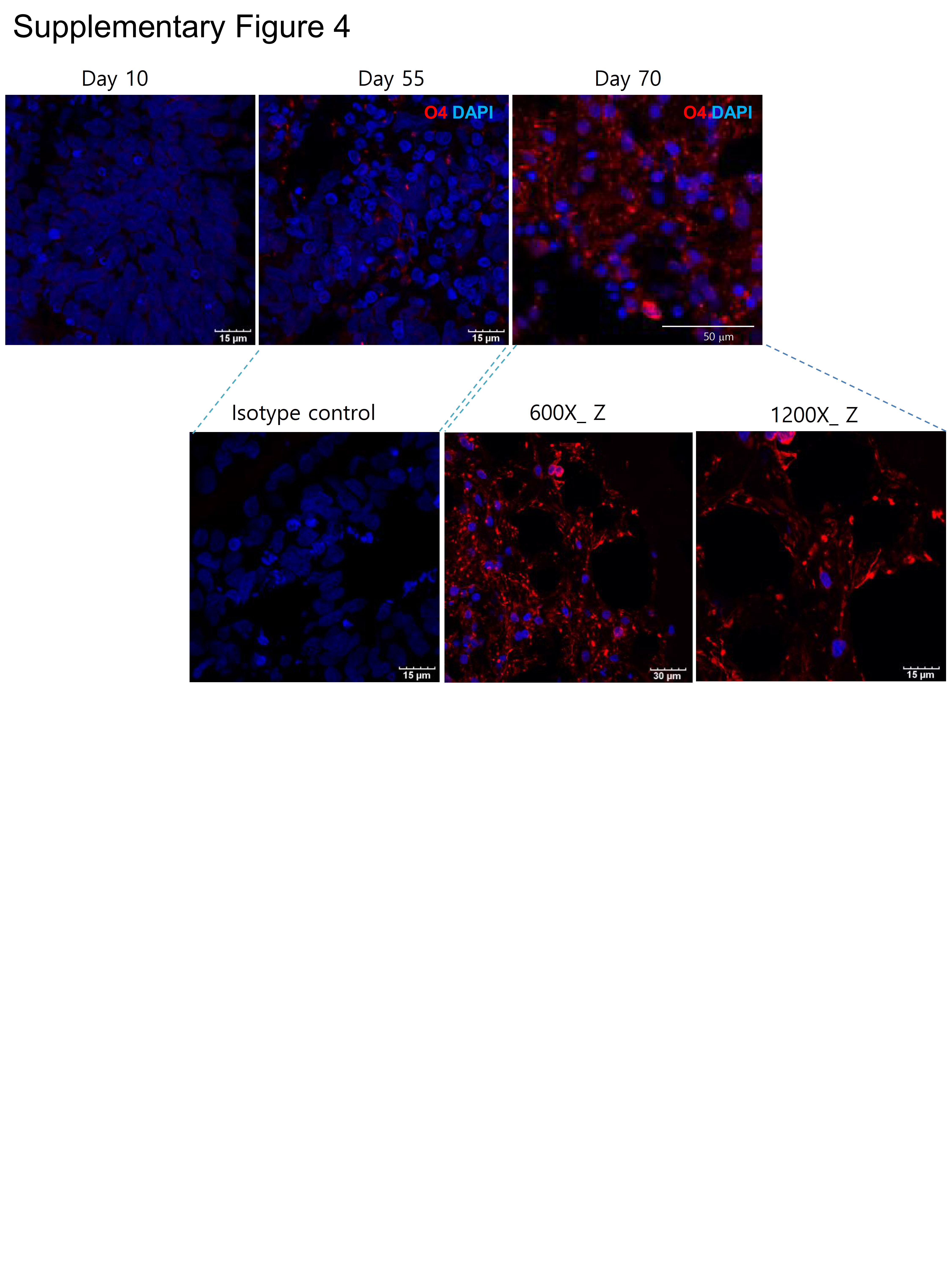

Supplement: Figure_S4.tif [file TEMI_A_1812435_SM2972.tif]

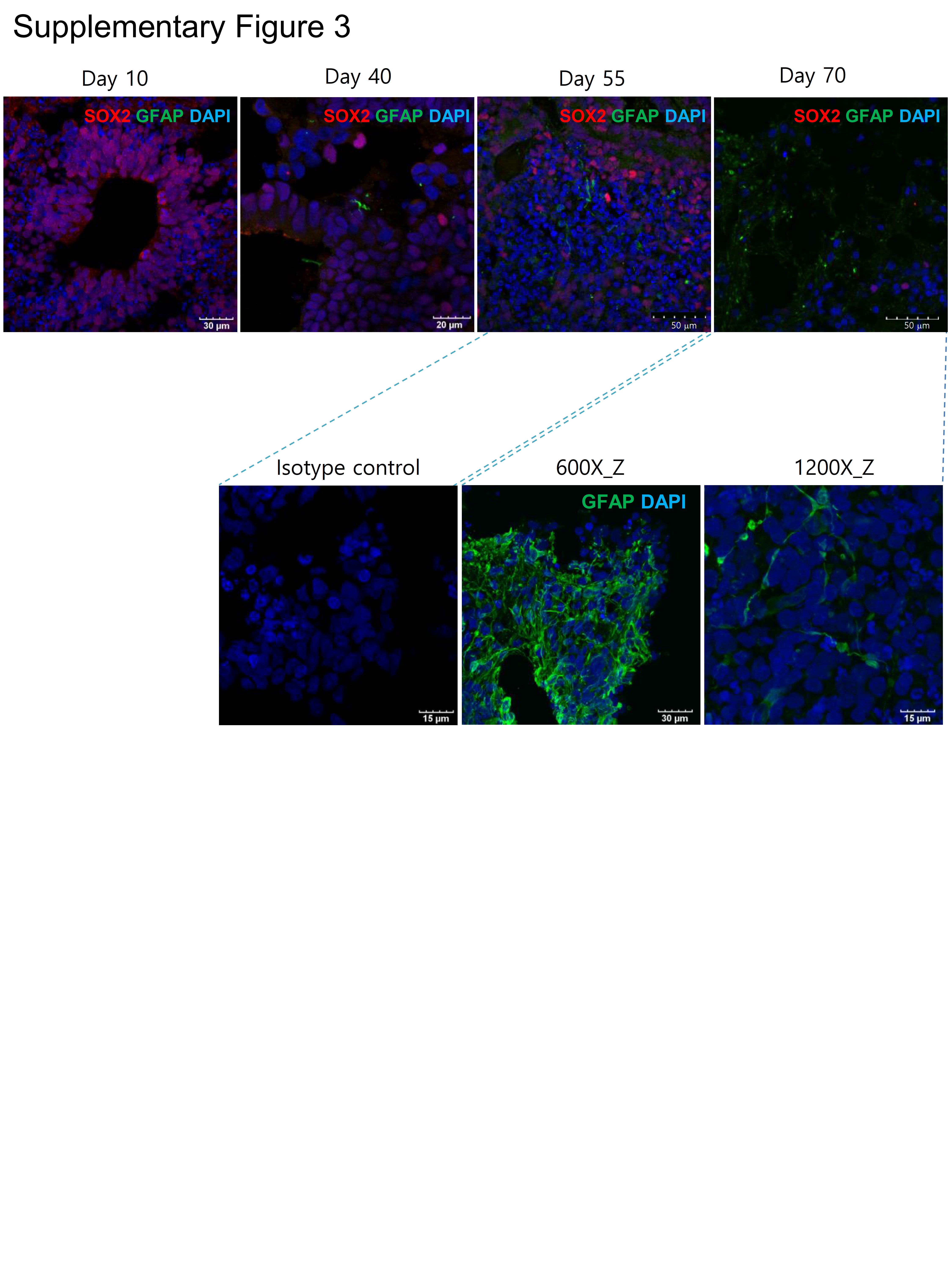

Supplement: Figure_S3.tif [file TEMI_A_1812435_SM2971.tif]

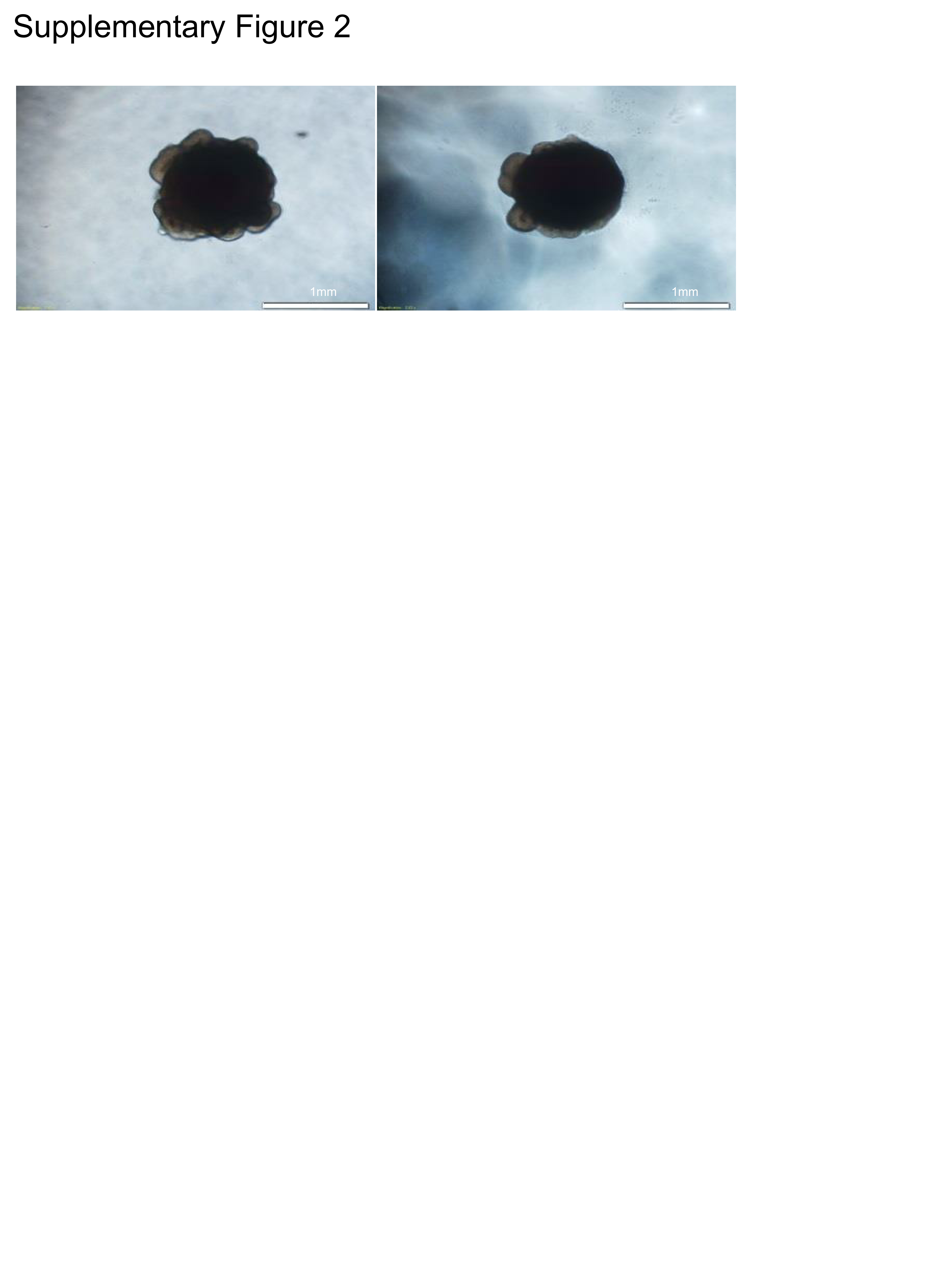

Supplement: Figure_S2.tif [file TEMI_A_1812435_SM2970.tif]

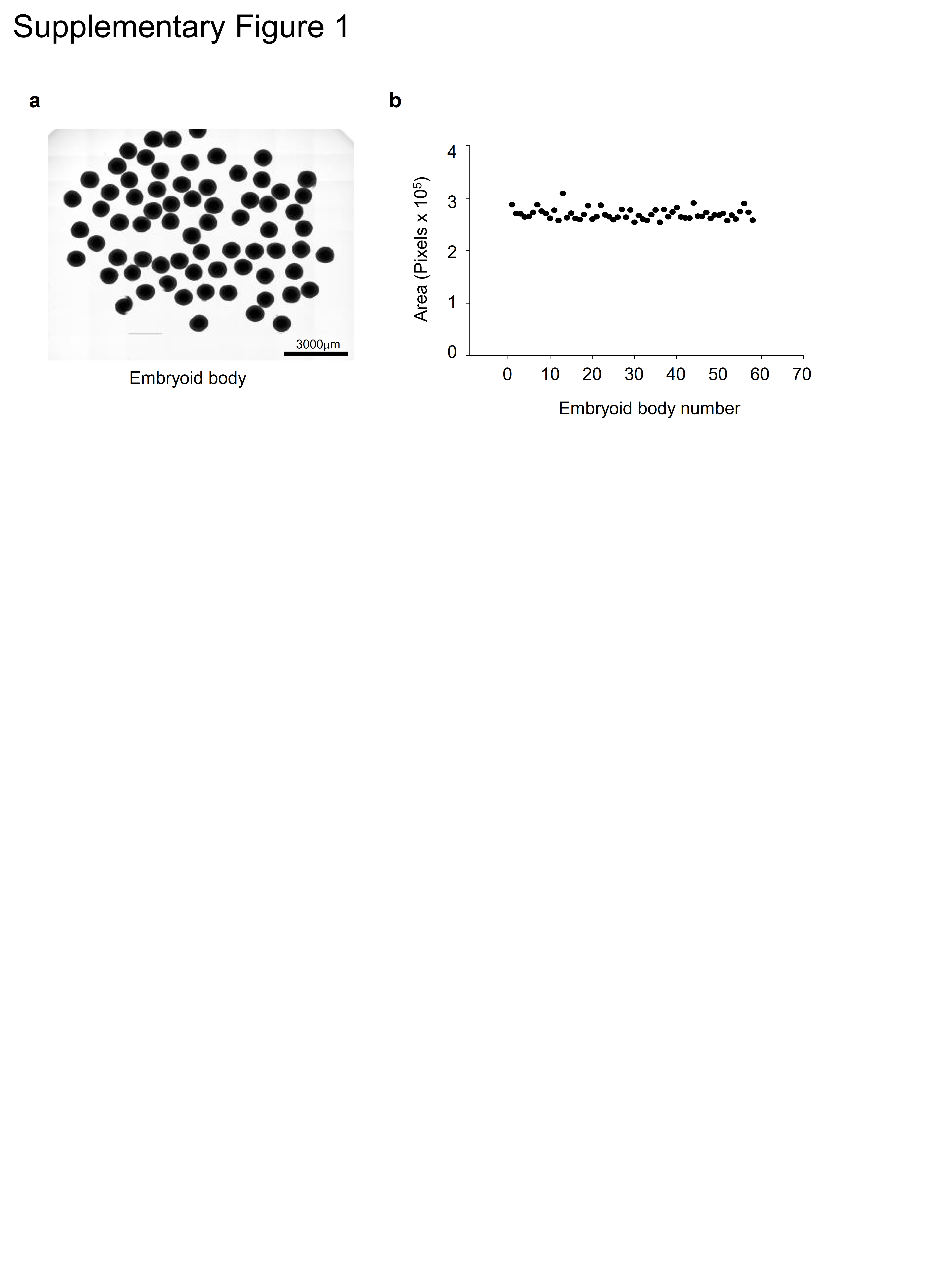

Supplement: Figure_S1.tif [file TEMI_A_1812435_SM2969.tif]
